# Epilepsy*Read before the Medical Club.

**Published:** 1884-07

**Authors:** Floyd S. Crego

**Affiliations:** Assistant Physician to Buffalo State Asylum. Lecturer on Diseases of the Nervous System in Medical Department, Niagara University


### BUFFALO

### Medical and Surgical Journal.

Vol. XXIII. JULY, 1884. No. 12.

### Original Communications.

### Epilepsy.*

Floyd S. Crego, M. D.,

Assistant Physician to Buffalo State Asylum. Lecturer on Diseases of the Nervous System in Medical Department, Niagara University.

In this paper I have attempted to give some of the most generally accepted theories regarding the disease and the methods of treatment, both medical and surgical, and have presented them largely in the language of the various authors quoted. These theories and methods have been brought together in the form of an article, for convenience of presentation in the most compact form for the busy practitioner.

" Epilepsy is a chronic functional disease of the nervous system, characterized by recurring paroxysms of impairment or loss of consciousness, accompanied, usually, by partial or general convulsions."—*Ross.*

This definition comprehends the following particulars :

1st. It is a functional disease.

2d. It is characterized by recurring paroxysms of impairment or loss of consciousness.

* Read before the Medical Club.

3d. It is generally, but not always, accompanied by convulsions.

These are essential points in the diagnosis of the disease. Epilepsy tends to become chronic, and the early recognition of it is, therefore, of the greatest importance.

Marshall Hall was the first to locate this disease in the brain. He considered the primary seat to be in the medulla, and that it had a peculiar effect on the nerves of the throat, so that at each attack there was produced a mild spasm of the muscles, which he called trachelismus, or a severe spasm which he called laryngismus. "It is a strange phenomenon," said he, "that these causes should select the muscles of the throat principally for the display of their influence." He went so far, and was so grounded in his belief, that he recommended, for the petit mal or slight spasm, venesection, and for the grand mal or severe spasm, tracheotomy. Brown-Sequard maintained that the primary seat of the disease was in the medulla, or, in many cases, in the base of the brain, and that the fit was produced by the action of carbonic acid gas or other toxic agent. Dr. A. Flint, Sr., sustains Brown-Sequard in the following language, as reported in the *New York Medical Journal*, that the manifestations of epilepsy were dependent upon a toxical agent of some kind, produced in the body in some manner, and in proof, advanced these points:

1st. In the absence of any generally received pathological doctrine, he assumed, as a postulate, that epilepsy had no established anatomical character. It might be associated with different lesions, and in a certain number of cases no lesion was discoverable.

2d. There was an analogy between the phenomena of epilepsy and those known to be produced by a toxical agent. The closest analogy existed between epilepsy and uræmic coma with convulsions. An attack of the latter might so closely simulate epilepsy that a diagnosis could not be made until the urine had been examined. The action of remedies which had been found useful, as the bromides and belladonna, might be explained by saying that they enabled the nervous system to tolerate, to a greater or lesser extent, the toxical agent on which the disease depended.

Kussmaul gave, as the result of his experiments, that the epileptic phenomena were due to an anæmia of the brain. This theory has many adherents, and Echeverria, in speaking of it, says: "Were I to present a definition embracing the principal features of the disease, I should say that epilepsy is a disease constituted by chronic paroxysms excited upon a direct or reflex action of the medulla oblongata in a condition of exalted irritability coincident with sudden depression in the cerebral circulation, and with loss of consciousness, with or without muscular spasms." This definition, he says, does not differ from that of Van Der Kolk, which seems to be the most rational. For various reasons, which he states, he further says: "I am led to admit that the medulla oblongata is the original seat of epilepsy, and that the disease primarily involves the vaso-motor nerves."

The theory of Hughlings Jackson is next to be considered. It is supported by Gowers, one of the most careful students of the disease. This theory holds that the phenomena of epilepsy are all explained by a discharging lesion of the cells of the gray matter, and that the disease affects the motor centers and does not often have its primary seat in the medulla. Gowers says: "There are no facts to warrant us in seeking the seat of the disease elsewhere than in the gray matter in which the discharge commences. This is, in most cases, within the cerebral hemispheres, probably, often, in the cerebral cortex, although, possibly, in some instances, lower down, and may be even in the medulla. Epilepsy is thus a disease of the gray matter, and has no uniform seat. It is a disease of tissue and not of structure." He also adds: "And the hypothesis of vascular spasm is as unneeded as it is unproven. The last question is: 'What is the nature of this tissue change? Can we form any opinion as to the character of the alteration in the gray matter which permits its sudden discharge?' It is answered in the following language: In the construction of a motor nerve center, it is requisite to create an apparatus which is always ready to evolve force, but which shall not do so spontaneously without the application of a stimulus of some kind or other, either physical or mental. The peculiarity of nerve cells is that they possess two qualities: They prepare material which, by undergoing oxidation, generates force, and yet, they can prevent this material from so acting, although blood is circulating all around it charged with oxygen. In states of spasm this property of the nerve cell is lost or much impaired, and the discharge of highly unstable cells leads to the discharge of healthy cells in other centers, with which there is an anatomical connection by fibres. The discharging lesion may be likened to a fulminate which overcomes the resistance of less unstable compounds. The nerve cells are likened to so many Leyden jars, which generate the electricity. Thus the explosion is due to a sudden diminution of resistance by which the pent-up nerve force is released, thus causing the epileptic phenomena."

This theory of Hughlings Jackson is one which affords, to many minds, a full and satisfactory explanation of the singular phenomena. The arguments, pro and con, I have not space to present. One very strong point in favor of this theory is that "when the commencement or aura was in the hand, a ligature to a certain point, as the elbow, would stop the fit."

The recent studies have been more beneficial than the explanation of the theory, as they have proven that much can be done to alleviate the disease and that many patients are saved who formerly were allowed to go on from bad to worse till death ended the scene.

We will now review the results of treatment, including both the action of drugs and the surgical means which have been found useful. We must remember that we have to deal with a chronic disease, and that the treatment must be persistent and long continued. It is a fair criticism to make of the treatment of epilepsy generally, that it is not begun soon enough, that it is not continued long enough, and is too readily interrupted upon a slight pretext, as the presence of an acne, or minor symptoms of indigestion. In this way a favorable result may be jeopardized.

"We have still too little definite knowledge of the pathology of epilepsy to permit of safe generalization regarding the possible mode of action of the several drugs which are found clinically useful. All that can be done is to recognize the kind of action which they have been found to exert upon the nervous system." The various bromides are found valuable, both as palliative and curative agents. All persons cannot tolerate them, and some are relieved by them, only, when associated with other remedies. The bromides, in their action, have been said to cause contraction of the small vessels of the brain, but it is exceedingly doubtful whether their influence in epilepsy is due to this action. "They have unquestionably a direct influence upon the nerve elements, diminishing, for instance, reflex action in the spinal cord, and act on the nerve cells of the brain, and it is probable that mental dullness and sleepiness, produced by large doses, are due to such action. If, then, we believe with Hughlings Jackson that the primary change in epilepsy is a morbid action of nerve cells, it is reasonable to conclude that the drug does good by directly influencing them."—*Gowers.*

In choosing which one of the bromides to use we should remember that it is an undoubted fact that the bromide of potassium has greater power to control this influence than any of the other bromides, notwithstanding it has less of free bromide than any of the other salts, as the bromide of lithium contains ninety-three per cent., bromide of ammonium, eighty-one per cent., bromide of sodium seventy-seven per cent., and the bromide of potassium sixty-nine per cent. It has been maintained that the bromide of sodium does not cause disturbances of the stomach so frequently as the bromide of potassium, but this is not borne out by clinical experience. The usual method employed in the administration of the drug, is a dose given at certain intervals during the day. The average dose for an adult is, grains xxx, three times a day, largely diluted with water, and given before meals. When the drug has been taken for a few days, reduce to gr. xx, three times a day. "A novel mode of determining the amount needed is to test the reflex action of the muscles of the fauces by touching them. If we find they do not readily respond, the patient is fully under control of the drug." The dose above mentioned should be continued from day to day, without any interruption, for weeks and months, unless severe toxic symptoms occur, when it should be reduced, but not suspended. Bromides are not readily eliminated from the system, and in this small dose we have the blood constantly saturated with the drug, and hence its beneficial effect. From such a dose we have no bad effects but a slight acne. which is readily relieved by the use of a few drops of liq. potass. arsenitis twice a day.

Many cases are relieved and many cured by the use of this drug alone. Of one of these cases the history is as follows: J. C., admitted June 2, 1883, aged 36, lawyer. No history of insanity, epilepsy or other nervous disease in the family. Patient, up to present attack, had been perfectly healthy. About one year since, after a hard day's work, had an attack, during which he lost consciousness for a few moments. A physician who was called said it was nothing serious, that he only fainted.

This was undoubtedly an attack of petit mal, and about five weeks ago he had the second attack, similar in character.

After several attacks his physician told him he had epilepsy. His attacks were finally accompanied with mania, lasting several hours, and on this account he was sent to the asylum.

On the day of admission he had an attack of petit mal. Was given bromide of potassium, grains xxx, three times a day, directed to exercise in the open air, and put on liberal diet. In two weeks the bromide was reduced to grains xx, t. d. At the end of six months patient had no unpleasant symptoms, except some gastric disturbance and constipation, both of which, however, he had been subject to from time to time for years. The former was readily relieved by confining him to an exclusively milk diet, the latter by use of rhamnus frangula. He had a few restless nights, which were relieved at once by a moderate dose of chloral at bed time. He continued to improve, and after a year's treatment, without the recurrence of an attack, was discharged, recovered.

The length of time a patient should go without a fit is from twelve to thirteen months, and medicine should be continued from eighteen months to two years.

At the end of this time the patient is said to be safe. Gowers recommends, in certain cases, when the fits recur at definite intervals, what is called the large dose method of giving bromides. He gives from ʒij to ʒiij, either just before the expected attack or during the day. This is given in a half a pint of water, and being so largely diluted does not cause either pain or vomiting. This dose may be increased to half an ounce, but not beyond the amount that produces temporary lethargy and mental dullness. Gowers has given six hundred and forty grains at one dose. This method has been so little used that it is difficult to tell what will be its practical value. Some cases do not tolerate the bromides alone, but these can take Brown-Sequard's mixture, the recipe for which is given in all works on epilepsy. In those cases where syphilis is suspected, iodide of potassium should be given with the bromides. The combinations of bromides with other drugs are of great value, as in many cases a better effect is produced by the combination than by either drug given alone. One of the best remedies to associate with the bromides is digitalis. It is especially recommended in those cases in which the disease is complicated by cardiac troubles, valvular defects, dilatation, irregularity and undue frequency, of the heart's action. It is also of considerable use in cases of nocturnal epilepsy.

In what way digitalis is useful in epilepsy is at present unknown, but Gowers reports cases relieved by this drug in combination with bromides when each separately had failed. Belladonna is associated with the bromides, and is said to be of value in cases of petit mal. Its influence is probably due to its direct action on the nervous system. This is highly recommended by English authors, and is also used by some specialists in this country. A case which I observed some four years since was permanently relieved by the use of atropia in 1-120 of a grain dose twice a day. Dr. Roberts Bartholow, in speaking of this drug, says: " The best results are obtained from it in nocturnal epilepsy, in petit mal, and in pale, delicate and anæmic subjects with cold hands and feet, blue skin and weak heart." He also says: " I have given strychnia with the bromides, in cases of epilepsy occurring in weak and anæmic subjects."

Fluid ext. ergot in doses of twenty minims and fluid ext. conium in same dose, associated with bromides, have been of great service and are highly recommended by some. The careful regulation of the diet, with plenty of air and exercise, are necessary adjuncts of treatment.

We see, then, from the foregoing that the bromides are the most useful remedies, and I present here some facts which may be of service in their administration:

First, the bromides generally succeed less well with children than adults, perhaps because the epilepsy of childhood is more allied to congenital states of the nervous centers than that of adult age. It is also more rapidly eliminated by children and the reflex action of the spinal cord with greater difficulty suppressed. Clark recommended brom. pot. grs. x to xx, daily, for a child one to three years of age. Between five and ten years, grs. xxx; ten and fifteen years, grs. xxxx to clxxx. "It appears from various sources and experiments that this drug is only readily absorbed when largely diluted and when the mucous membrane is healthy. Hence in dyspepsia and gastritis it should be avoided. Though this drug is a fixed salt, it is disturbed by acids, hence it should be given before meals when the secretion of the stomach is neutral, for when the stomach is at work and contains food, the secretion is acid. It is apt, under these circumstances, to disturb the stomach and to cause free bromine to be liberated, and gastric irritation and eructations are produced. It is absorbed readily by the rectum, and should be given in a great amount of water, as grs. x to ℥j. The irritant qualities of bromides forbid the hypodermic use."

"An ordinary dose shows itself in the blood in about ten minutes and is eliminated in eight hours. A large dose is not eliminated for twenty-four. Hence a large dose once a day or a small dose three times a day keeps the blood charged with the drug constantly. Diuretics should be regularly given in order to increase the urinary secretion and the elimination of bromide. This will prevent certain cutaneous diseases that are very disagreeable to the patient. Iron should always be associated with the use of bromides if there be the slightest appearance of anæmia, which its long continued use is apt to induce. Hitherto electricity has been used with comparative infrequency. Understanding, as we now do, that this is a disturbance of the cells of brain tissue, manifested by an explosive discharge, we have the *rationale* of the treatment of epilepsy by electricity, and our efforts are directed toward the epileptic change in the brain."

Althaus, in his new treatise, recommends the transverse galvanization through the mastoid processes and galvanization of the sympathetic. Erb follows this idea and employs a stable, but very feeble current of four to six elements, which he passes through the head obliquely from the temporal region and upper part of the forehead on one side to the opposite side of the back of the neck. He allows the poles to remain from one-half to one minute, then he employs it longitudinally from the forehead to the back of the neck. Where vaso-motor symptoms are marked during an attack, he galvanizes the cervical sympathetic. Careful trial of faradic currents to the head appears justifiable. Althaus advises the galvanic current to be applied to peripheral nerve tracts in which the aura is situated. He would also use it if a neuralgia, a nerve injury, a cicatrix or the like is suspected as the starting point of the epilepsy, even if it is not the site of an aura; reports some striking results. Erb has had some favorable cases which should lead us to faithfully use this remedy.

We next consider the surgical treatment of epilepsy, which presents to us several operations:

1st. Trephining.

2d. Ligature of carotid.

3d. Nerve stretching.

Trephining is a very old operation, but before the present century was little used, and there were few recoveries. In the beginning of the present century Tissot had good success in this operation. Dr. Dudley, of the Transylvania University, was the first to introduce it into this country. His writings were brilliant, but much of the glory was taken away when a colleague, jealous of his success, translated many of the Doctor's sayings from articles written by Mon. Tissot. Since that time this operation has been performed by many with a more or less degree of success. Nearly all of the authors agree that the "principal indication for resorting to trephining is when the disease follows an injury to the head, from which we conclude that a depression, a fracture of the internal table, a spicula of bone or a collection of fluid is pressing upon and irritating the brain." The plainest indication for the use of the trephine is actual depression of the cranial bones at the seat of the injury, for here "the cause of the morbid condition of brain is not only apparent, but its precise location indicated." We find, first, in the history of this disease, that the records of medicine in our own county look upon the operation, especially when the cause is traumatic or when there is depression, as justifiable. Dr. Stephen Smith, in 1852, in the *New York Medical Journal*, reports 27 cases, and considers the operation simple, and from which we could hope much in cases where we have depression as the known cause of the disease. In a private letter written me a few days since, he says: "In 1852 I tabulated 27 cases, which was the first article I knew of on this subject at that time. Since then the operation has been very frequently performed, and it is a very proper operation when there has been a local injury. It is a very harmless operation if cautiously performed. I should select cases by preference which have a cicatrix which is tender or the seat of an aura. Since 1852 I have operated upon or been consulted in ten cases, as recorded below.

Dr. Agnew says: "In most cases, under favorable circumstances the operation is not only justifiable but urgently demanded. The danger from this is very much exaggerated." He considered the conditions which justify the operation in cases following fractures, "when the occurrence of very frequent attacks causes progressive weakening of mental faculties, and when remedies have failed to control the disease." Dr. Gross has had poor success in this operation and fails to give his opinion as to the advisability of performing it. Dr. Echeverria, of New York, who has written extensively, records 148 cases and says: "I need not add that from my own experience in the matter and from analysis of cases, I am in favor of trephining the skull for relief of epilepsy, due to local injury to the head, and I consider, with Dr. Van Buren, that the operation is comparatively a simple and harmless one, its seriousness being explained by the fact that it is performed in cases which, from their nature, are fatal. The risks of trephining are greatly diminished if especial care be taken not to injure the dura mater and cerebral membranes."

We find, then, these three noted surgeons and one specialist in favor of this treatment. By referring to Ashurst's Surgery we find, with regard to trephining: "I can only state that I consider the operation unavoidable; its risks are not inconsiderable. When we remember the well-known fact that epilepsy is apparently and temporarily curable by various remedies which have at least the merit of being harmless, we should pause before we recommend an operation which may not, improbably, itself cause death, and in which the proportionate benefits are doubtful." Ericsen says: "The operation and its results are certainly not favorable." Dr. Russel considers it justifiable and has good success. Heath, of London, reports several favorable cases. Billroth and Luecke report 49 per cent. of recoveries in this operation and favor it. Dr. Adolph Bardelaben, of Berlin, says, in his recent work: "I consider it only justifiable when the person's life is in danger." Nothnagel, in Ziemssen's, says: "We are willing to grant that the operation, with our present experience, is generally allowable, and we must regard its success in any particular case as a piece of good fortune which could not have been predicted."

Table showing the results in one thousand and sixty-five cases:

| | Cases. | No. Cured. | No. Died. | Not Relieved. | Relieved. |
|---|---|---|---|---|---|
| Billroth & Luecke.... | 709 | 352 | 357 | .. | .. |
| Echeverria .......... | 148 | 93 | 28 | 9 | 18 |
| Walsham ............ | 26 | 20 | .. | 6 | .. |
| Russel .............. | 50 | 24 | 6 | 10 | 4 |
| Billings............. | 72 | 42 | 16 | 10 | 4 |
| Smith, in 1852........ | 27 | 7 | .. | 15 | 5 |
| Smith, in 1884........ | 10 | 3 | .. | 3 | 4 |
| Operated in this city by three surgeons... | 3 | 1 | 2 | .. | .. |
| Volkman's Klinick.... | 14 | 11 | 3 | .. | .. |
| Also with no depression | 8 | 7 | 1 | .. | .. |
| | 1,067 | 560 | 413 | 53 | 35 |

The table presented above gives as cured about one-half of the cases. This is about the average which is recorded in other tables. This is, then, an operation which we should try in appropriate cases before giving up our patient as lost. With the medical and surgical means at our command we certainly can place this disease among the list of curable ones, instead of consigning it to the category of the incurable and hopeless.

